# Thermal Ablation for Colorectal Liver Metastases: Current Evidence and Future Horizons

**DOI:** 10.3390/diagnostics16142239

**Published:** 2026-07-17

**Authors:** Xinliang Liu, Wenlong Qiu, Zhiguang Hu, Qian Liu

**Affiliations:** 1 Department of Surgical Oncology and General Surgery, The First Affiliated Hospital of China Medical University, Shenyang 110001, China; liuxinliang@cmu.edu.cn; 2 Department of Colorectal Surgery, National Cancer Center/National Clinical Research Center for Cancer/Cancer Hospital, Chinese Academy of Medical Sciences and Peking Union Medical College, Beijing 100021, China; 15154117801@163.com; 3 Department of Ultrasound, National Cancer Center/National Clinical Research Center for Cancer/Cancer Hospital, Chinese Academy of Medical Sciences and Peking Union Medical College, Beijing 100021, China; guguaiguang@163.com

**Keywords:** colorectal cancer, liver metastasis, thermal ablation, radiofrequency ablation, microwave ablation

## Abstract

Liver metastasis constitutes the principal cause of mortality in colorectal cancer, with about 50% of patients developing liver metastasis during disease progression. While surgical resection remains the cornerstone of curative-intent treatment, thermal ablation is rapidly reshaping the landscape of local tumor control in well-selected individuals. This comprehensive review analyzes the evolving role of thermal ablation in the management of resectable, unresectable, and recurrent disease, with the aim of establishing contemporary best practices and identifying critical frontiers for future research.

## 1. Introduction

Colorectal cancer (CRC) has consistently posed a significant challenge to global public health systems [1]. China has a large population base, and the incidence and mortality rates of CRC are higher than those in other parts of the world [2]. Anatomically, venous blood from the colorectum flows into the liver via portal circulation, making the liver the most important target organ for CRC hematogenous metastasis. It has been reported that approximately 45% of CRC patients eventually develop liver metastasis, with about 20% presenting with liver metastasis at the time of initial diagnosis—referred to as synchronous liver metastasis—and an additional 25% developing liver metastasis following surgery for CRC, known as metachronous liver metastasis [3]. Liver metastasis is a major cause of death among CRC patients [4]. Therefore, standardized treatment of liver metastases is the key to prolong the survival prognosis of patients.

Hepatic resection (HR) is now widely recognized as the best treatment for colorectal liver metastasis (CRLM), and patients with CRLM who undergo radical resection have a five-year survival rate of 50–70% [5]. Unfortunately, radical resection is feasible in less than 25% of patients with CRLM due to excessive tumor burden, poor anatomical location of the tumor, proximity to important vascular or biliary structures, combinations of extrahepatic disease, or other comorbidities [6,7,8]. This substantial treatment gap has catalyzed the evolution of thermal ablation (TA) techniques, particularly radiofrequency ablation (RFA) and microwave ablation (MWA). Due to its characteristics of less destruction of healthy liver parenchyma, lower complication rate, and shorter operation time and hospitalization time, TA is gradually being viewed as an important local treatment method—applicable as a curative-intent option for unresectable CRLM, as an intraoperative adjunct to resectable disease, or as a salvage measure for recurrent lesions [9]. Compared with stereotactic body radiotherapy (SBRT), TA offers advantages in repeatability, shorter procedure time, and lower cost for lesions ≤3 cm, whereas SBRT may be preferred for tumors adjacent to hollow viscera or when percutaneous access is challenging [10]. Both modalities, however, must be integrated with systemic therapy and tailored by a multidisciplinary team (MDT) encompassing colorectal surgeons, hepatobiliary surgeons, medical oncologists, interventional radiologists, and radiation oncologists to optimize sequence and combination based on tumor genetics, liver function, and prior treatment responses [11,12].

The past decade has witnessed three pivotal shifts in TA application: transition from purely palliative to curative-intent therapy, integration with systemic treatments in hybrid regimens, and emergence of standardized ablation margin criteria, which has allowed TA to show good oncological results and gain recognition in CRLM treatment. This comprehensive review examines the evolving role of TA across resectable, unresectable, and recurrent disease states, with the aim of establishing contemporary best practices and identifying critical frontiers for future research.

## 2. Literature Search Strategy

Although this narrative review is not a systematic review, we adopted a systematic literature search strategy to ensure comprehensiveness and scientific validity. The databases searched included PubMed, Embase, Web of Science, Cochrane Library, and ClinicalTrials.gov. The search covered the period from database inception to 2025. The keyword combinations comprised “thermal ablation”, “radiofrequency ablation”, “microwave ablation”, “colorectal liver metastases”, “CRLM”, “liver metastasis”, and their corresponding MeSH terms, with adjustments made as appropriate for each database.

Regarding literature selection and prioritization, we gave preference to: (1) high-level evidence, including randomized controlled trials (RCTs) and large-sample propensity score-matched studies; (2) systematic reviews and meta-analyses published within the last ten years; and (3) the most recent clinical guidelines and expert consensus documents issued by internationally recognized authorities, including the NCCN guidelines, the COLLISION trial group multidisciplinary consensus document, and the Guidelines for the Diagnosis and Comprehensive Treatment of Colorectal Liver Metastases. In addition, we manually cross-referenced the bibliographies of included articles to supplement relevant studies.

## 3. Technical Evolution of TA

### 3.1. RFA

RFA is the most widely used TA modality in clinical practice [13]. It works by inserting a radiofrequency electrode needle into the tumor area under imaging guidance, forming a complete circuit in the patient’s body through an external grounding pad, and releasing an alternating current with a frequency between 350 and 500 kHz to drive high-frequency oscillations of ions, resulting in localized hyperthermia (about 60 °C) that causes coagulative necrosis of tissues in a short period of time [14]. However, when the temperature reaches or exceeds 100 °C, water boiling and evaporation cause tissue dehydration and subsequent carbonization. The carbonized tissue acts as a poor conductor, hindering the further transmission of radiofrequency current [15]. Once the carbonized tissue completely encases the exposed tip of the electrode needle, the transmission of radiofrequency current is entirely interrupted, thereby terminating the ablation process. This phenomenon is referred to as “roll-off” [16]. Another important factor influencing the ablation zone is capillary blood perfusion. The heat dissipation effect near blood vessels limits the temperature rise in perivascular tissue, thereby compromising the ability of RFA to achieve complete ablation in areas adjacent to large vessels. This phenomenon is known as the “heat-sink effect” [17]. Affected by “roll-off” and the “heat-sink effect”, RFA in clinical practice is associated with the following limitations: (1) inability to achieve complete ablation in a single session, (2) restricted ablation volume, and (3) incomplete tumor ablation.

To overcome these adverse effects, researchers have made systematic and comprehensive improvements, which have demonstrated favorable efficacy in tumor treatment. These strategies include: (1) Intermittent energy delivery based on real-time temperature or impedance monitoring to avoid excessive tissue carbonization and delay the onset of “roll-off”. Examples include real-time MRI thermometry-guided systems [18], fiberoptic thermocouple-based real-temperature monitoring [19], and impedance-modulated systems that improve predictability in heterogeneous tissues [20]. (2) Electrode needle enhancements, such as multi-tined expandable electrodes, which enable a larger ablation zone in a single application [21], and hybrid cryo-RFA systems that combine rapid freezing and thawing cycles [22]. (3) Heat-sink mitigation, including pharmacological agents to slow vascular flow [23], inflatable balloons [24], transcatheter embolization [25], and trans-arterial chemoembolization [26].

### 3.2. MWA

Microwave ablation (MWA) utilizes microwave antennas or probes to emit high-frequency electromagnetic waves, typically operating at 915 MHz or 2450 MHz, which excite polar molecules (e.g., water molecules) within the tissue to rotate and vibrate. This induces friction and collision between molecules, thereby elevating tissue temperature and promoting coagulative necrosis [27]. Compared with RFA (Table 1), MWA generates a more homogeneous and larger ablation zone, reducing the time required for tumor ablation [28].

**Figure 1:**
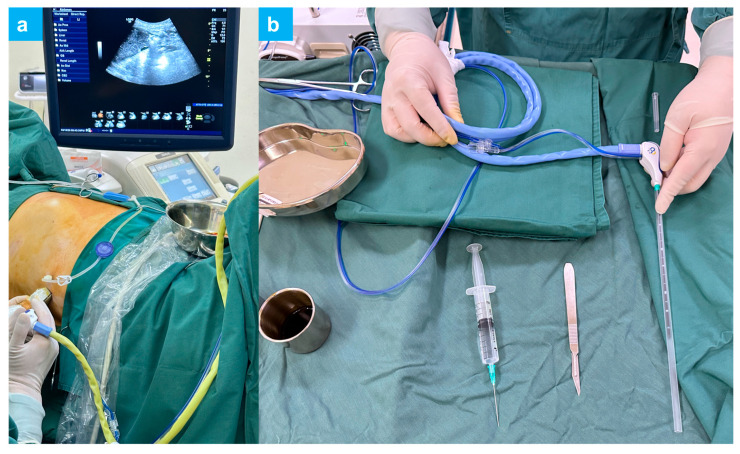
Clinical application of TA, illustrated with percutaneous MWA. (**a**) Intraoperative ultrasound-guided percutaneous MWA for liver metastases. (**b**) Preoperative preparation for ultrasound-guided percutaneous MWA.

MWA’s advantages over RFA stem from several key factors: higher intratumoral temperatures (90–120 °C vs. 60–100 °C for RFA) [29], a reduced heat-sink effect in perivascular tumors [30], and faster ablation times (5–10 min vs. 12–20 min for RFA) [31] (Figure 1). However, MWA also has certain deficiencies, including: (1) thermal injury to normal tissue adjacent to the tumor; (2) limited ablation volume, with effective ablation confined to solid tumors up to approximately 5 cm in diameter [32]; and (3) an immunosuppressive tumor microenvironment following ablation [33].

With advances in materials science, research into sensitized MWA has progressively deepened. Nanoparticle-enhanced ablation using gold-silica nanoshells [34], microcapsules with loading capacity [35], ionic hydrogels [36], tumor agglomeration materials based on metal–organic frameworks [37], and organic materials with a lumen structure [38] have all been shown to exert a sensitizing effect on MWA.

These innovative materials achieve sensitization primarily through three mechanisms: expanding the ablation zone, reducing thermal damage to normal tissues, and activating antitumor immunity [39]. Although these approaches are still at the stage of animal testing, they hold promise for future clinical application.

## 4. TA for Initial Unresectable CRLM

Since the 1990s, with continuous advancements in ablation technology, TA—particularly RFA and MWA—has been progressively adopted in the treatment of CRLM. Its advantages include minimal invasiveness, efficacy, safety, and reproducibility, especially for patients who are ineligible for HR due to unfavorable tumor location, presence of extrahepatic disease, or insufficient future liver remnant volume. These aspects have been addressed in relevant studies and guidelines both domestically and internationally [40,41,42,43].

### 4.1. Definitive Oncological Benefits

Owing to its favorable safety profile and high local control rate, multidisciplinary guidelines and expert consensus groups generally recommend TA for small-sized unresectable CRLM (≤3 cm) [10,11,44]. However, several studies have found that the efficacy of TA is restricted by tumor size, decreasing exponentially for lesions larger than 3 cm [45,46,47]. It has been estimated that the risk of local progression increases by 22% for every 5 mm increment in CRLM diameter [48]. In contrast, a systematic review by Nieuwenhuizen et al. [49] reported that TA is safe and can induce long-term disease control for unresectable intermediate-sized CRLM (3–5 cm). Similarly, Dijkstra et al. [50] found that TA for intermediate-sized unresectable CRLM is safe and achieves long-term local control in the vast majority of patients. Because the efficacy of SBRT is independent of vascular proximity and largely unaffected by lesion size or anatomical accessibility, it has been proposed as an alternative to TA for perivascular, subdiaphragmatic, or larger CRLM [51,52]. Consequently, the results of the COLLISION-XL trial (NCT04864435)—a Phase II randomized controlled trial evaluating local ablative methods for unresectable CRLM of 3–5 cm—are awaited to further clarify the role of ablation for intermediate-sized unresectable disease [53].

For unresectable CRLM, multiple RCTs have demonstrated that combined local and systemic therapy confers a survival benefit over either modality alone [11,54]. The prospective EORTC-CLOCC trial further demonstrated a significant overall survival (OS) benefit for RFA plus chemotherapy over chemotherapy alone, with 8-year OS rates of 35.9% versus 8.9% [55]. Additionally, Kong et al. [56] reported that in patients with initially unresectable CRLM, RFA combined with systemic chemotherapy plus targeted therapy as first-line treatment significantly prolonged both progression-free survival (PFS) and OS. Faiella et al. [57] demonstrated the safety and efficacy of combined trans-arterial embolization and MWA for the treatment of large unresectable hepatic metastases (maximum diameter >3 cm).

Taken together, these findings suggest that for unresectable CRLM of suitable size and location, TA combined with other therapies may offer the greatest clinical benefit.

### 4.2. Bridging to Resection

In principle, the indiscriminate use of TA in CRLM treatment should not be encouraged, as surgeons should always strive for margin-negative resection. However, most patients with CRLM are not candidates for HR at the time of diagnosis. Specific scenarios may justify the use of TA, including the intention of parenchymal preservation, downstaging as part of a two-stage treatment strategy, or management of patients at high risk for major liver resection [58]. Under such circumstances, we recommend adding TA while closely monitoring patients for early recognition of local treatment failure. For instance, Imai et al. [59] found that RFA combined with HR achieved outcomes comparable to HR alone in a propensity score-matched analysis. Another study reported that RFA plus HR resulted in poorer survival than HR alone; however, in patients with four or more tumors, long-term survival rates were similar between the two groups [60]. Moreover, other results suggest that neoadjuvant TA enables conversion to resectability in 23–31% of patients with initially unresectable CRLM [61,62,63]. The ongoing COLLISION-XL trial is prospectively comparing MWA versus SBRT as bridging therapies, with interim analysis showing superior resection rates with MWA (29% vs. 17%, *p* = 0.03) [53].

**Figure 2:**
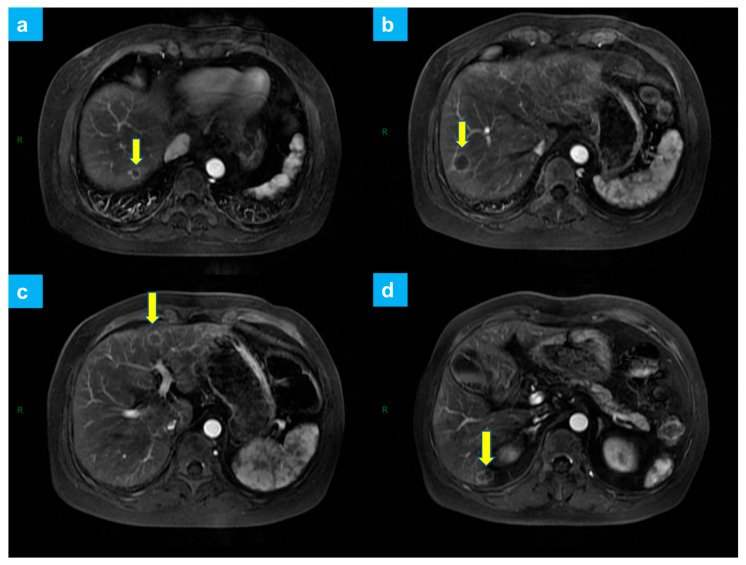
A case of TA for unresectable CRLM. Before TA, the enhanced MRI showed obvious ring-shaped enhancement in the arterial phase (yellow arrow). (**a**–**d**) show the distribution of liver metastases from four different sites in one patient.

**Figure 3:**
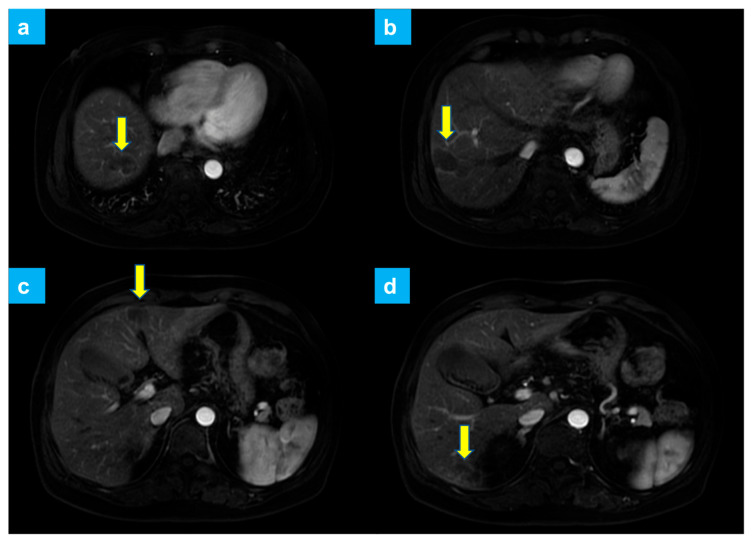
The case shown in Figure 2. Two months after TA, the enhanced MRI showed no obvious enhancement on the scan (yellow arrow). (**a**–**d**) show the imaging changes of liver metastases in four sites of the patient shown in Figure 2 after TA.

Therefore, TA and HR should not be mutually exclusive. In appropriate patients, TA may convert initially unresectable CRLM to a state amenable to HR, thereby improving patient survival outcomes (Figure 2 and Figure 3).

## 5. TA in Resectable CRLM

Although various domestic and international guidelines recommend TA as an important option for local curative therapy in patients with CRLM, TA is typically employed as an alternative or adjunctive treatment for patients with unresectable disease who refuse surgery, or for those who are poor surgical candidates. To date, there is no consensus on whether TA can be used as first-line therapy for patients with resectable CRLM who are eligible for both HR and TA [41,42,43,64,65].

### 5.1. Challenging Surgical Paradigms

**Figure 4:**
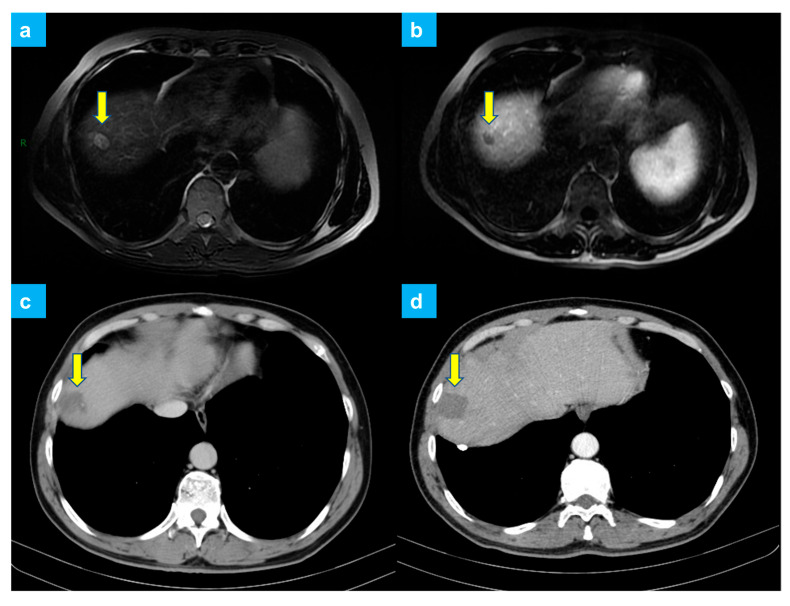
A case of TA for initially resectable CRLM. (**a**,**b**) Before TA, the enhanced MRI showed a slight ring enhancement (yellow arrow). (**c**,**d**) Forty days after TA, the enhanced CT showed that the ablation lesion in the upper part of the liver did not exhibit any significant enhancement (yellow arrow).

Several retrospective studies have compared survival, recurrence, and safety outcomes between HR and TA in patients with CRLM. Wang et al. [66] analyzed 210 patients with ≤3 liver metastases (maximum diameter ≤5 cm) treated with either TA or HR. The results showed no statistically significant difference in OS between the two groups. Although median disease-free survival (DFS) was longer in the HR group than in the TA group (22 months vs. 14 months), there was no significant difference in DFS between the two groups for patients with metastases ≤3 cm. In a retrospective study [67] of 2367 patients who underwent radical treatment for CRLM between 2008 and 2018 at Sun Yat-sen University Cancer Center, no statistically significant difference in OS was found between HR and TA groups for patients with ≤5 tumors with a maximum diameter of ≤5 cm, although HR demonstrated a better local control rate. Subgroup analysis, however, showed that for patients with a maximum diameter ≤3 cm, there was no statistically significant difference in local progression-free survival between the two groups. Similarly, Tawara et al. [68] compared the efficacy of TA versus HR for CRLM and found that HR was more effective overall, but no survival difference was observed between the two groups for metastases ≤2 cm. Collectively, these findings suggest that the efficacy of TA versus HR for resectable CRLM is not equivalent (Figure 4).

High-quality RCTs are the gold standard for validating treatment efficacy. An earlier attempt to compare TA with HR was terminated prematurely due to treatment preferences and misconceptions about eligibility (LAVA, ISRCTN52040363) [69]. The ongoing NEW-COMET trial (NCT05129787) [70] compares the 12-month local tumor progression rate after TA versus HR. The HELARC trial (NCT02886104) [40] is designed to compare simultaneous versus staged resection (primary tumor then percutaneous TA) for CRLM. Furthermore, the international Phase III randomized controlled COLLISION trial [71] was stopped early after meeting predefined criteria for early benefit. The trial demonstrated a high likelihood (conditional power >90%) of proving non-inferiority regarding OS, non-inferior local control, and fewer complications with TA compared with HR for small-sized CRLM (≤3 cm).

Taken together, these studies indicate that the efficacy of TA is closely linked to the size of the metastatic tumor: the smaller the tumor, the relatively better the ablation outcome. For resectable CRLM, HR remains the reference approach, while TA may be considered in selected patients with small lesions, high surgical risk, parenchymal preservation needs, or favorable tumor biology (Table 2).

### 5.2. Clinical and Economic Considerations

Beyond oncological outcomes, TA offers tangible advantages in safety, recovery, and resource utilization. The COLLISION trial reported significantly fewer adverse events (19% vs. 46%) and shorter median hospital stay (1 vs. 4 days) compared with HR, with zero treatment-related mortality versus three deaths in the surgical arm [71]. A multicenter target trial [75] emulation of 1334 patients with solitary CRLM ≤5 cm confirmed lower major complication rates (2.1% vs. 5.0%) and reduced median costs ($4820 vs. $10,239) for ablation. These findings are consistent with a systematic review and meta-analysis demonstrating a 74% relative risk reduction in major complications for TA [76]. Formal quality-of-life and comprehensive cost-utility data are still limited but are being actively collected in ongoing trials; their results are expected to further inform patient-centered and value-based decision-making in the future.

### 5.3. Ablation Margin—A Critical Technical Parameter

Compared with HR, resectable CRLM have a higher recurrence rate after TA [77,78]. Local tumor recurrence rates after RFA have been reported to range between 4 and 40% [79,80], whereas the corresponding range for MWA is 6–10% [44,81]. Predictors of local tumor recurrence after TA include tumor size and ablation margin [82,83]. As described above, a maximum metastasis diameter >3 cm is associated with recurrence [67,84,85]. The ablation margin is even more important because it is the only parameter that surgeons can control.

#### 5.3.1. Evidence

Previous studies found that an ablation margin less than 5 mm is a risk factor affecting recurrence after TA therapy [58,86]. Data from large observational trials report that an ablation margin of 5 mm is the minimum requirement for acceptable local tumor control [87]. However, by pooling evidence from 21 studies comprising 2005 participants and 2873 ablated CRLM lesions, Chlorogiannis et al. [88] found that a minimal ablation margin >5 mm is the minimum critical endpoint required, whereas a margin of at least 10 mm yields optimal local tumor control after TA. Moreover, a study by Wang et al. [48] showed local tumor progression-free survival rates of 74% when margins were 6–10 mm and 80% when margins exceeded 10 mm. Therefore, in the much-anticipated prospective COLLISION trial, the researchers conducted a corresponding analysis of the ablation margin. The results showed that in the TA group, the imaging-based A1 rate (<5 mm margins) for ablated tumors was 17 (5%) of 322, of which eight (47%) recurred during follow-up. Consequently, imaging-based A0 ablations (≥5 mm margins) correctly reflected the absence of local tumor progression in 290 (95%) of 305 ablated target tumors [71]. These findings indicate that when TA is performed for resectable liver metastases of appropriate location and size, the ablation margin should be at least 5 mm, provided that surrounding tissues and organs are not damaged.

#### 5.3.2. Measurement

Accurate margin assessment requires 3D software-based quantification rather than subjective two-dimensional visual comparison of pre- and post-ablation images [89,90]. A multicenter retrospective study [91] of 400 ablated CRLM lesions demonstrated that when a minimum ablative margin ≥5 mm was confirmed using biomechanical deformable image registration and artificial-intelligence (AI)-based auto-segmentation, the 3-year local disease progression rate was 0–2% across institutions. Notably, intraprocedural CT-based minimal ablation margin quantification has been shown to significantly outperform the 4–8-week follow-up CT in predicting local tumor progression, emphasizing the importance of real-time intraprocedural assessment. Advanced techniques such as deformable image registration and deep-learning-based auto-segmentation are increasingly available and facilitate precise, reproducible margin measurement [92].

#### 5.3.3. Imaging Protocol for Margin Assessment

The current standard recommends immediate post-ablation contrast-enhanced CT as the primary imaging modality for margin evaluation [89,93]. Pre-procedural triphasic thin-slice liver CT is the minimum requirement for tumor delineation and ablation planning; for sub-centimeter lesions (<1 cm), hepatobiliary contrast-enhanced MRI is recommended due to its superior detection sensitivity [94,95]. The same 3D software-based assessment should be repeated at the 4–8-week post-ablation contrast-enhanced CT to confirm technical efficacy [96]. This dual-timepoint approach—intraprocedural for immediate technical success confirmation and follow-up for durability assessment—is endorsed by the 2026 multi-society Delphi consensus on liver tumor ablation margin assessment, which represents the first global framework standardizing margin measurement, interpretation, and reporting [97].

#### 5.3.4. Management Strategies When Adequate Margins Cannot Be Achieved

When intraprocedural 3D assessment reveals a minimal margin <5 mm, a tiered approach is recommended. First, repeat ablation within the same session should be performed whenever technically feasible and safe [98]. A prospective multicenter study [98] evaluating 3D-confirmed margin assessment in CRLM ablation defines a minimal ablation margin ≥5 mm as the necessary condition for technical success and mandates same-session repeat ablation for margins below this threshold. If same-session completion is not possible, rescue ablation within 30 days is recommended to achieve a sufficient margin. For margins in the 5–10 mm range, biopsy of the ablation zone margin may be considered; if pathology confirms absence of residual viable tumor, this can provide additional confidence in local control [99]. Finally, when anatomical constraints—such as proximity to major bile ducts, the gastrointestinal tract, or the diaphragm—preclude achievement of a safe margin despite optimal technique, alternative modalities including SBRT or surgical resection should be considered [53,71]. This algorithmic approach ensures that margin assessment is not merely a descriptive endpoint but an actionable intraprocedural quality metric that directly informs treatment decisions.

## 6. TA in Recurrent CRLM

Despite complete tumor eradication, approximately 64–85% of patients develop new metastases after initial local treatment of CRLM [100,101]. Recurrence after radical treatment of CRLM can be classified into three scenarios: (1) local recurrence—new lesions at the original ablation site; (2) intrahepatic recurrence—new lesions in non-ablated portions of the liver; and (3) systemic recurrence—new lesions outside the liver, either alone or combined with new intrahepatic lesions [102,103].

### 6.1. Efficacy Evaluation

Studies report that the recurrence rate after local radical treatment of CRLM is significantly elevated [84]. Moreover, patients who experience recurrence after local radical therapy may face challenges such as insufficient future liver remnant volume [104]. Therefore, standardized management of recurrent CRLM is of critical importance.

**Figure 5:**
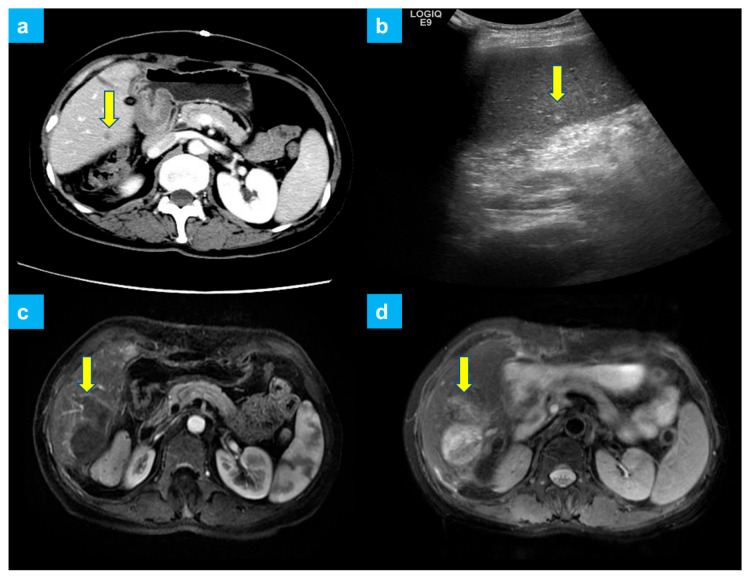
A case of TA for recurrent CRLM. (**a**,**b**) One month after resection of multiple intrahepatic metastases, ultrasound and enhanced CT showed a new low-density nodule in the right posterior lobe of the liver, with blurred edges, raising suspicion of metastases (yellow arrow). (**c**,**d**) Two months after TA, the enhanced MRI showed an abnormal mass signal shadow in the surgical area of the right lobe of the liver. No obvious enhancement was found on the enhanced scan (yellow arrow).

For extrahepatic recurrence, systemic therapy is essential. For intrahepatic recurrence, both HR and/or TA are considered the standard of care according to current literature and international guidelines [41,42,43,105]. Although repeat HR is relatively safe and feasible, it can be technically challenging due to adhesions and reduced liver volume following prior surgery [106]. Given the superior safety profile of TA and the fact that TA is less affected by previous surgical injury, it has been questioned whether TA could represent a safer and equally effective alternative to repeat HR for small-sized recurrent lesions. In response, Dijkstra et al. [107] extracted data from the prospectively maintained AmCORE CRLM database and found that repeat TA was not statistically different from repeat HR with respect to OS, DFS, local tumor progression-free survival, and complications. The authors concluded that TA should be considered as a valid and less invasive alternative to HR for small-sized (0–3 cm) recurrent CRLM (Figure 5). Previous research on outcomes of repeat HR and repeat TA also support these findings [108,109].

### 6.2. Complementary Role of (Neo)adjuvant Systemic Therapy

Furthermore, recurrent disease is associated with micrometastatic disease and dormant cancer cells, which are not addressed by repeat local treatment alone [110]. This suggests a higher risk profile, for which aggressive systemic therapy in addition to local treatment is recommended [111]. A recent pooled meta-analysis showed a trend toward improved survival with the addition of neoadjuvant systemic therapy to repeat local treatment [112]. The largest registry study to date (LiverMetSurvey) also found an OS benefit favoring neoadjuvant systemic therapy before repeat local treatment: a 5-year OS of 61.5% versus 43.7% (*p* = 0.028) [113]. However, the EORTC 40983 trial by Nordlinger et al. [114] and the JCOG 0603 trial by Kanemitsu et al. [115] showed no benefit from adding (neo)adjuvant systemic therapy for resectable and/or ablatable disease after initial local treatment of CRLM.

To definitively evaluate the value of neoadjuvant systemic therapy prior to repeat local treatment in patients with recurrent, locally treatable CRLM, the Phase III multicenter randomized controlled COLLISION RELAPSE trial [116] (Table 3) has been initiated. If the addition of neoadjuvant systemic therapy to repeat local treatment proves superior to repeat local treatment alone, this approach may prolong life expectancy and increase disease-free survival, albeit at the cost of potential systemic therapy-related side effects.

## 7. Future Directions

As a key locoregional modality in the comprehensive treatment of CRLM, TA is evolving from a simple thermal destruction technique into a precision treatment platform that integrates image guidance, energy control, tumor biology, and immune microenvironment modulation. By continuously addressing clinical pain points, conducting rigorous research, and promoting technological convergence, TA is expected to provide safer, more effective, and cost-effective treatment options for a broader patient population, ultimately improving the overall treatment landscape of CRLM. Future directions can be summarized in the following aspects, each of which has received preliminary exploration and support from cutting-edge research. To provide readers with a balanced perspective, we stratify the following directions according to their current stage of evidence development: (1) advanced imaging and margin assessment (closest to clinical translation, with ongoing multicenter trials); (2) AI (promising but requiring validation in large multicenter studies); (3) circulating tumor DNA (ctDNA)-guided monitoring (emerging clinical data with prognostic value, awaiting routine validation); (4) immunotherapy combinations (predominantly preclinical and early-phase, with uncertain clinical benefit); and (5) lessons learned from hepatology regarding multimodal integration (a reference template for future post-ablation surveillance of CRLM).

### 7.1. Refinement and Innovation of the Technology

To overcome complex lesions, resolve traditional challenges such as marginal residue caused by the heat-sink effect and incomplete ablation of irregular large tumors, and pursue predictable and visualizable radical ablation, future efforts will rely on intelligent upgrades of energy platforms. The next generation of MWA can produce a more stable and spherical ablation zone through optimized antenna design, showing the potential to outperform RFA for tumors adjacent to large blood vessels, thereby helping to overcome the heat-sink effect [13,117]. Closed-loop AI control systems that integrate real-time temperature and impedance feedback aim to achieve individualized accurate prediction and regulation of the ablation zone. Deep integration of image navigation and efficacy evaluation represents another key direction. Post-procedural assessment increasingly relies on hepatobiliary-specific contrast-enhanced MRI, whose functional imaging sequences can detect residual active tumor tissue earlier and more sensitively, and which is considered the gold standard for evaluating local treatment efficacy [118]. For lesions adjacent to critical structures, irreversible electroporation has emerged as a key complementary modality due to its non-thermal mechanism and the advantage of preserving vascular and biliary structures [119,120].

### 7.2. Optimization and Expansion of Treatment Strategies

To clarify the clinical positioning of TA in CRLM, elucidate the competitive relationship between ablation and surgery, and maximize the synergistic effect between TA and systemic therapy, future decisions will extend beyond anatomical considerations. They will integrate tumor biological characteristics and ctDNA dynamics to build predictive models for accurate selection of the best-benefiting population [121,122]. In terms of treatment strategy, the spatiotemporal synergy between ablation and systemic therapy is a highly discussed topic. After effective conversion therapy with neoadjuvant chemotherapy, ablation can serve as a key local consolidation method. Even more promising is the combination of ablation and immunotherapy to treat CRLM. Theoretically, TA can activate systemic antitumor immunity through immunogenic cell death; however, the optimal timing and mode of combination with immune checkpoint inhibitors remain to be defined by large-scale clinical trials [123,124,125]. Currently, the continuous generation of high-level evidence is critical. Regarding the core controversy of whether ablation is non-inferior to surgery for resectable CRLM, there is an urgent need to design more rigorous multicenter Phase III randomized controlled trials.

### 7.3. Improvement of the Efficacy Evaluation System

Establishing a dynamic, multi-dimensional system for prognostic monitoring and complication prevention and control is a crucial step toward earlier and more precise assessment of treatment effectiveness and reduction in serious complication risks. Therefore, it is necessary to develop a dynamic evaluation system that goes beyond purely morphological assessment. With functional imaging at its core, combined with a dynamic monitoring network incorporating liquid biopsies (ctDNA), this system can facilitate early detection of minimal residual lesions and systemic recurrences, thereby enabling precision follow-up for CRLM [126]. At the same time, it is essential to build a system for the proactive prediction and standardized prevention and control of complications. For risks such as liver abscess and biliary tract injury, predictions should be made by integrating patient-related, tumor-related, and procedural factors [102,127]. Furthermore, preventive use of antibiotics, thermal monitoring techniques, and alternative interventions such as irreversible electroporation should be applied in a standardized manner.

### 7.4. Improvement of Diagnosis and Treatment Paradigm

To ensure that each CRLM patient receives the most individualized and rational treatment sequence, the application of TA should be embedded within a value-oriented, multidisciplinary integrated diagnosis and treatment framework. Ablation treatment decisions must be made within a high-quality MDT setting. Future MDTs will dynamically formulate strategies based on real-time shared multimodal data [128]. Moreover, study endpoints should be expanded from a sole focus on survival duration to a comprehensive “value-based medicine” measure that includes patient-reported outcomes and health economics evaluations, thereby fully capturing the real benefits of treatment for CRLM patients [129].

### 7.5. Multimodal Post-Ablation Surveillance: Lessons from Hepatology

The monitoring of patients after CRLM ablation shares conceptual parallels with surveillance in chronic liver disease—both require integration of imaging, biomarkers, and structured clinical follow-up to detect disease progression at an early, treatable stage. In this context, Ichim et al. [130] recently demonstrated the value of combining elastography-based imaging biomarkers, serum biomarkers, and clinical assessment for monitoring disease progression in alcohol-associated cirrhosis. While the underlying disease is distinct from CRLM, the methodological framework—multimodal integration of quantitative imaging (elastography), circulating biomarkers, and structured clinical follow-up—offers a valuable template for CRLM post-ablation surveillance.

## 8. Conclusions

In summary, CRLM represents both a central focus and a major challenge in the treatment of CRC. For the management of CRLM, HR and TA each have their own advantages and limitations. For CRLM that is not amenable to surgery alone, TA combined with systemic therapy—when appropriately indicated—can offer patients a chance at curative treatment, which is superior to palliative chemotherapy alone. For resectable liver metastases, TA does not completely replace HR; however, for patients with few and small-diameter lesions, TA may achieve outcomes comparable to those of HR. For recurrent CRLM, repeated TA treatment may prolong patient survival.

Nevertheless, the efficacy of TA requires further validation through high-quality RCTs. More importantly, in the management of oncologic diseases, a holistic perspective is essential. Treatment decisions should be based on a systematic evaluation of patient needs, with personalized treatment goals and the implementation of corresponding comprehensive therapies to improve patient survival and prognosis. Through continuous technological innovation, in-depth mechanistic research, and rigorous clinical validation, TA is expected to provide safer, more effective, and more cost-effective treatment options for a broader population of patients with CRLM.

## Figures and Tables

**Table 1:** Comparison of RFA and MWA.

Performance Parameter	RFA	MWA
Energy delivery mechanism	Resistive heating via ionic agitation (350–500 kHz)	Dielectric heating via water molecule rotation (900–2450 MHz)
Heat generation pattern	Outward from electrode tip; requires tissue conductivity	Volumetric around antenna; conductivity-independent
Ablation time per lesion	Longer: 12–20 min for 3–4 cm target	Shorter: 5–10 min for similar size
Ablation zone shape	Irregular, ovoid	Spherical, ellipsoid
Max ablation diameter (single probe/antenna)	~3 cm	~5–6 cm
Susceptibility to heat-sink effect	High	Low
Effect of tissue charring	Increases impedance (“roll-off”)	No impedance limitation
Intraprocedural pain	More painful	Less painful
Grounding pads required	Yes	No
Risk of skin burn/tract seeding	Lower risk	Slightly higher risk
Lesion size	<3 cm: Good efficacy; 3–5 cm: Higher risk of incomplete ablation; >5 cm: Not routinely recommended.	<3 cm: Equally effective; 3–5 cm: More complete ablation due to higher thermal efficiency; >5 cm: Not routinely recommended.
Lesion location	Perivascular: Efficacy compromised by significant heat-sink effect; Peribiliary: Lower biliary complication rate, relatively safer.	Perivascular: Less affected by blood flow, better efficacy than RFA; Peribiliary: Biliary complications significantly higher than RFA.

**Table 2:** Comparison of factors favoring TA versus HR.

Factor	Favoring TA	Favoring HR
Tumor size	≤3 cm (optimally)	>3 cm
Tumor number	≤5	Any number, if resectable
Location [72]	Deep-seated (≥3 cm from surface)	Superficial (<3 cm from surface)
RAS/BRAF [73,74]	Wider margins for mutant patients	No difference in outcome
Extrahepatic disease [71]	None	May still be resected if controlled
Comorbidities	High risk, poor surgical candidate	Fit for surgery

**Table 3:** Summary of major completed and ongoing clinical trials evaluating TA in CRLM.

Trial	Design	Population	Primary Endpoint	Status
COLLISION	Phase III RCT, non-inferiority	Resectable small-size CRLM	Overall survival	Completed
NEW-COMET	Phase III RCT, non-inferiority	Resectable CRLM	Local tumor recurrence	Ongoing
HELARC	RCT	Resectable CRLM	Efficacy/safety of local ablation	Ongoing
COLLISION-XL	Phase III RCT	Unresectable intermediate-size CRLM	local tumor progression-free survival	Ongoing
COLLISION RELAPSE	Phase III RCT, superiority	Recurrent locally treatable CRLM	Overall survival	Ongoing
EORTC-CLOCC	Phase II RCT	Unresectable CRLM	Overall survival	Completed

## Data Availability

No new data were created or analyzed in this study. Data sharing is not applicable to this article.

## Abbreviations

The following abbreviations are used in this manuscript:

AI	Artificial intelligence
ctDNA	Circulating tumor DNA
CRC	Colorectal cancer
CRLM	Colorectal liver metastases
DFS	Disease-free survival
HR	Hepatic resection
MDT	Multidisciplinary team
MWA	Microwave ablation
OS	Overall survival
PFS	Progression-free survival
RFA	Radiofrequency ablation
RCT	Randomized controlled trial
SBRT	Stereotactic body radiotherapy
TA	Thermal ablation
